# Cohort profile: Guangzhou breast cancer study (GBCS)

**DOI:** 10.1007/s10654-024-01180-y

**Published:** 2024-12-16

**Authors:** Jiao Wang, Na Li, Cheng Kun Xiao, Shu Shu Han, Min Jie Lu, Xiao Yi Lin, Ze Fang Ren, Lin Xu

**Affiliations:** 1 School of Public Health, Sun Yat-Sen University, 74 Zhongshan 2nd Road, Guangzhou, Guangdong Province China; 2 School of Public Health, The University of Hong Kong, Hong Kong, China; 3 Institute of Applied Health Research, University of Birmingham, Birmingham, UK; 4 Greater Bay Area Public Health Research Collaboration, Guangzhou, China

**Keywords:** Breast cancer, Cohort, Case-control, Immunohistochemistry

## Abstract

The Guangzhou Breast Cancer Study (GBCS) is a patient-based prospective cohort study designed to identify risk factors and underlying mechanisms for breast cancer (BC) incidence and prognosis, specifically addressing the need for individualized prevention in South China, where BC incidence is notably high. Based in Guangzhou, China, the GBCS began recruitment in 2008, comprises three complementary studies: the Guangzhou breast cancer cohort with 5471 breast cancer patients, a case–control study with 1551 cases and 1605 controls, and an immunohistochemistry (IHC) cohort with 1063 breast cancer patients. Participants are primarily aged 41–60 years. Cohort follow-up is conducted every three months in the first year, every six months in the second and third years, and annually thereafter. High follow-up rates have been achieved until 2023, with 73.5% for the Guangzhou breast cancer cohort and 98.6% for the IHC cohort still active. Baseline data collection included demographic characteristics and breast cancer risk factors, while follow-up data included survival, treatment details, disease history, occupational history, post-diagnostic lifestyle, and laboratory measures, including genetic markers, proteins, and environmental exposures. The study encourages global collaborations and invites interested researchers to contact the corresponding author at xulin27@ mail.sysu.edu.cn with specific research ideas or proposals.

**Supplementary Information:**

The online version contains supplementary material available at 10.1007/s10654-024-01180-y.

## Why was the GBCS set up?

Breast cancer (BC) is the most prevalent cancer and the leading cause of cancer mortality in women globally [1], and its burden has been rising over the past decades [2]. As of 2022, the World Health Organization (WHO) reported 2.3 million new BC cases and 670,000 related deaths worldwide [3]. Although BC can affect women at any age after puberty, its incidence increases with age and significant geographical disparities exist, with higher rates in developed regions compared to less developed areas [4].

In China, the most populous developing country, rapid economic growth over the past five decades has brought about significant lifestyle and environmental changes, contributing to an increased BC burden. Currently, BC is the most common cancer in Chinese women, account for 12.2% of all new BC cases and 9.6% of BC-related deaths globally [5]. South China, in particular, shows the highest incidence of BC in the country [6], underscoring the need for region specific strategies to mitigate this rising burden. Furthermore, despite the development of various BC risk prediction models [7, 8], there is limited models tailored for Asian women that incorporate DNA or protein biomarkers [9]. Additionally, efficient prognostic information is needed to optimize patient care and alleviate the strain on healthcare systems.

**Fig. 1:**
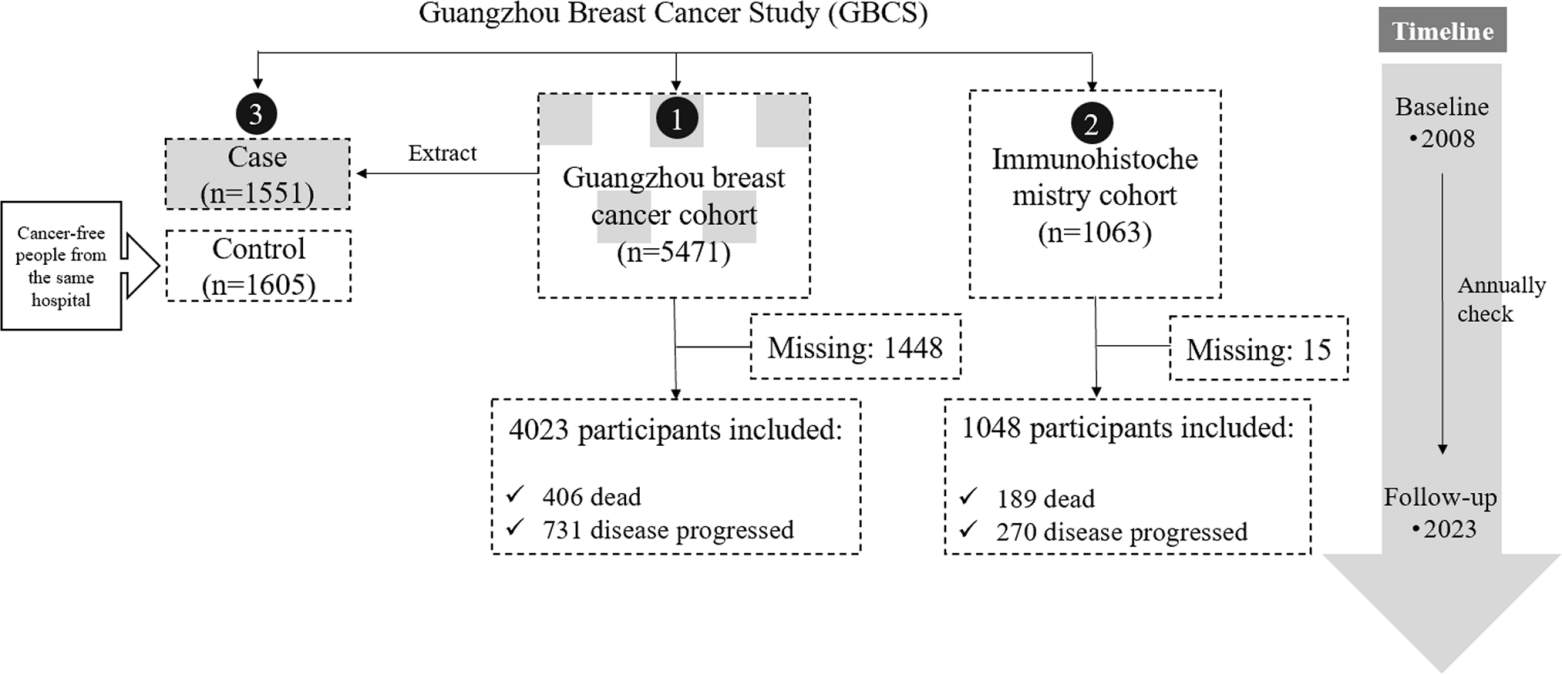
Overview of the Guangzhou breast cancer study (GBCS)

To address these challenges, the Guangzhou Breast Cancer Study (GBCS) was established in 2008 with the support of the School of public health, the First and the Second Affiliated Hospitals and the Cancer Center of Sun Yat‐sen University. The GBCS recruited 9029 patients with breast diseases, including 5471 BC patients for the Guangzhou breast cancer cohort. The whole study also includes a case–control study with 1551 BC cases and 1605 controls, as well as an immunohistochemistry (IHC) cohort including 1063 BC patients (Fig. 1). The GBCS aims to elucidate the risk factors and underlying mechanisms of BC incidence and prognosis, ultimately benefiting the local population and contributing to global BC research.

## Who is in GBCS?

The GBCS consists of three interrelated studies: a Guangzhou breast cancer cohort, a case–control study, and an immunohistochemistry (IHC) cohort, each designed to investigate different aspects of BC in South China.

### Guangzhou breast cancer cohort

The Guangzhou breast cancer cohort is a patient-based, prospective cohort study designed to explore the risk factors and prognosis of BC in South China. Participants were recruited from the First Affiliated Hospital, Second Affiliated Hospital and Cancer Center of Sun Yat-sen University from October 2008 to January 2018. Initially, 9029 patients with various breast diseases were enrolled in the study, all of whom provided informed consent (Supplementary Table 1**)**. Among these participants, 5471 were confirmed to have BC and met the inclusion criteria for the Guangzhou breast cancer cohort, which included: (a) a pathological diagnosis of primary BC; (b) newly diagnosed cases; and (c) residency in the Pearl River Delta region for at least five years. Exclusion criteria were applied to exclude patients with a history of malignancy or mental illness, those unable to communicate due to severe illness, and those with cognitive impairments. Of these 5471 BC patients, 335 were diagnosed with ductal carcinoma in situ and 5136 had invasive BC.

### Case–control study

In the GBCS case–control study focuses on identifying risk factors for the occurrence of BC. From October 2008 to March 2012, 1736 women diagnosed with BC from Guangzhou breast cancer cohort (as mentioned above) were unselectively included as cases. Moreover, cancer-free controls, frequency-matched by age, were selected from women undergoing routine medical examinations at the same hospitals during the same period. The inclusion criteria for controls included the absence of breast malignancy, confirmed by mammography, B-ultrasonography or self-reported, and residency in the Pearl River Delta region for at least 5 years. After excluding individuals diagnosed with other cancers, those with mental illness, and those who declined to complete questionnaires or donate blood samples, the final study included 1551 cases (89.3% participation rate) and 1605 controls (90.5% participation rate).

### Immunohistochemistry (IHC) cohort

The GBCS IHC cohort investigates the underlying mechanisms of BC progression. This cohort includes 1063 women diagnosed with primary invasive BC, with tumors larger than 1 cm, recruited from the Cancer Center of Sun Yat-sen University between January 2008 and December 2015. Exclusion criteria included a history of other malignant tumors or mental illnesses (such as Alzheimer’s disease) and severe illnesses or language difficulties that prevent completion of the questionnaire. Histone modification markers and protein levels in tumor and adjacent tissues were assessed using tissue microarrays (TMAs) and immunohistochemistry.

### How is the cohort followed up?

The GBCS cohort employs both active and passive follow-up methods. Active follow-ups are conducted every three months during the first year after diagnosis, either via phone calls or outpatient visits. In the second and the third years, follow-up occur every six months. Thereafter, participants are followed up annually until death. These follow-ups collect vital information, including survival statuses, treatment details, disease history, occupational history, post-diagnostic lifestyle changes, and updated contact details. Detailed follow-up variables are shown in Table 1. Passive follow-up is conducted through the hospital’s electronic medical record system, which provide authoritative data on disease progression and mortality. This method enables the capture of comprehensive clinical information, such as serum tumor markers, cell types and lipid profile, which are meticulously retrieved from medical records. As of 31 December 2023, at least four rounds of follow-up have been completed for all patients, with 4023 (73.5%) participants of Guangzhou breast cancer cohort and 1048 (98.6%) participants of IHC cohort being successfully followed up.

**Table 1:** Summary of follow-up variables in the Guangzhou Breast Cancer Study (GBCS) cohort

Sections	Variables
Survival statuses	Recurrence
	Metastasis
	Death
	Other newly diagnosed diseases
General treatment	Radiotherapy
	Chemotherapy
	Surgery
	Endocrine therapy
Disease history	Heart disease, hypertension, diabetes, allergies, hyperlipidemia, arthritis, other diseases
Occupational history	Type of occupation, daily working hours
Physical exercise	Type of activity, weekly activity hours
Personal health behaviour	Smoking status
	Tea
	Sleep habits
Serum tumor markers	CEA
	CA199
	CA125
	CA153
	CA211
Cell types	Eosinophil
	Basophil
	Lymphocyte
	Monocyte
	Neutrophil
Lipid markers	Total cholesterol
	Triglyceride
	High-density lipoprotein
	Low-density lipoprotein
Demography	Name
	Age
	Identity card number
	Address
	Mobile phone number
	Email Address
	Household size
	Menopausal status

### What has been measured?

The GBCS collected a comprehensive range of data through structured questionnaires, clinical records and laboratory assays to investigate BC etiology, progression and outcomes. All GBCS participants completed a structured questionnaire developed by the Breast Cancer Epidemiology Research Group of Vanderbilt University, tailored to reflect current research on BC etiology and the demographic characteristics specific to the Guangdong region. Baseline data were obtained by face-to-face interviews, typically lasting 30–60 min [10–12], and included information on demographic factors, menstrual and reproductive history, disease history, contraceptive use, hormone therapy, dietary history, physical activity, occupational history, personal habits, and family history of cancer. Detailed baseline characteristics of the participants from the questionnaire are shown in Table 2. Furthermore, clinical data, such as tumor size, nodal status, clinical stage, and receptors status (i.e., estrogen receptor (ER), progesterone receptor (PR), and human epidermal growth factor receptor 2 (HER2)), were meticulously retrieved from medical records. Participants fasted for 10–12 h before providing blood and urine samples, which were collected under the supervision of trained research assistants and stored at − 80 °C until further analysis. Each participant was assigned a unique identity number to ensure the accuracy and traceability of the samples.

**Table 2:** Summary of questionnaire data collected at baseline in the Guangzhou breast cancer cohort, IHC cohort, and case–control study

Exposure category	Variable/exposure
Past medical history	Diagnosed breast diseases (specifically itemized)
	Diagnosed tumor
	Diagnosed other diseases (specifically itemized)
	Infection-induced fever
	Dysmenorrhea
Menstrual and reproductive history	Age of menarche
	Parity and breastfeeding history
	Menopause status and associated timing
	Contraceptive history
Contraceptive methods and hormone use	Contraceptive history
	History of hormone-replacement therapy use
Physical activity	Physical activity at different ages
Dietary history	Meat
	Taste
	Oils
	Fried food
	The old fire soup
	Herbal tea
Personal health behaviour	Smoking
	Passive smoking
	Alcohol
	Tea
Occupational history	Occupation
	Afternoon nap
	Daily sleep time
	Occupational exposures
Family history	Family history of cancers
Development history and measurement	Height
	Weight
Demography	Name
	Age
	Identity card number
	Address
	Mobile phone number
	Email Address
	Occupation
	Household size
	Marital status
	Educational level
	Income

A wide range of laboratory variables was measured, including 26 urinary metals, 53 single nucleotide polymorphisms (SNPs), 27 cytokines, 14 histone modification markers, 16 proteins, 4 Epstein-Barr virus (EBV) antibodies, anti-*Toxoplasma gondii* (*T. gondii*) and anti-*Chlamydia trachomatis* (*C. trachomatis*) IgG. Each laboratory variable and the corresponding measurement sample are shown in Table 3.

**Table 3:** Summary of laboratory variables measured in the Guangzhou breast cancer cohort, IHC cohort, and case–control study

Variable categories	Variables	Populations
Urinary metals	Batch 1: beryllium, titanium, germanium, niobium, molybdenum, tellurium, platinum, bismuth, vanadium, chromium, cobalt, arsenic, selenium, rubidium, strontium, cadmium, indium, cesium, and thallium Batch 2: lithium, manganese, nickel, copper, zinc, barium, lead, vanadium, chromium, cobalt, arsenic, selenium, rubidium, strontium, cadmium, indium, cesium, and thallium	Batch 1: 240 cases and 246 controls Batch 2: 445 cases and 495 controls
SNPs	rs1136410, rs17883901, rs1800871, rs1801133, rs1805087, rs1805414, rs2051579, rs2069705, rs2228611, rs2234693, rs250108, rs28366003, rs2981582, rs3219145, rs3736360, rs3918242, rs6505162, rs8679, rs10889221, rs1078985, rs12644365, rs4976412, rs6926191, rs9485372, rs9383951, rs2471214, rs10107389, rs10511591, rs3176626, rs11033111, rs2046210, rs14192, rs911157, rs1052536, rs1061217, rs1075496, rs1290005, rs1711418, rs1800796, rs2273534, rs2292179, rs2605039, rs3751812, rs3816358, rs3845744, rs6829064, rs8109631, rs10216653, rs10989563, rs11078676, rs12380505, rs17102086, and rs2591592	1551 cases 1605 controls
Histone modification markers	H3K4me2, H3K4me3, H3K9me1, H3K9me2, H3K9me3, H3K27me3, H3K36me3, H4K20me3, H3k9ac, H4k5ac, H4k8ac, H4k12ac, H4K16ac, and H3K18ac	1061 patients of IHC cohort
Proteins	MCM5, FOXA1, ECM1, NDUFAB1, AGR2, Eelfg, Glut, STMN1, Calpain7, CMBL, HID1, ENO1, MLPH, ERK1, MIPEP, and GRB7	1061 patients of IHC cohort
Cytokines	MIP1-β, IL-6, IFN-γ, IL-1ra, IL-5, GM-CSF, TNF-α, RANTES, IL-2, IL-1β, Eotaxin, Basic-FGF, VEGF, PDGF-BB, IP-10, IL-13, IL-4, MCP1, IL-8, MIP1-α, IL-10, G-CSF, IL-15, IL-7, IL-12p70, IL-17, and IL-9	794 cases 268 controls
EBV antibodies	IgA antibodies against EBV VCA-p18 and EBNA-1, IgG antibodies against EBV VCA-p18 and EBNA-1	349 cases 500 controls
Anti-*T. gondii* IgG	-	1121 cases 400 controls
Anti-*C. trachomatis* IgG	-	1121 cases 400 controls

The study was in accordance with the Declaration of Helsinki and approved by the Sun Yat-sen University Ethics Committee (Institutional Review Board approval number: 2012–8).

### Urine metal detection

Twenty-six metals were quantified using inductively coupled plasma mass spectrometry (ICP-MS) (Agilent 7500ce ICP-MS, Agilent Technologies). Before analysis, urine samples were diluted with dilute nitric acid. The quantification of metals was performed by ICP-MS, calibrated with external standards provided by Spex Industries, with internal standards (89Y, 103Rh and 175Lu) added to each sample to ensure accuracy [13]. Samples underwent at least three replicate analyses, with rigorous quality control, including matrix blanks and quality-control samples, to avoid cross-contamination and ensure precision.

### DNA isolation and genotyping

Genomic DNA was extracted from blood samples using the TIANamp Genomic DNA Kit (TianGen Biotech Co., Ltd., Beijing, China). Fifty-three SNPs were genotyped using Sequenom’s MassARRAY system (San Diego, California, USA) [14, 15]. Both positive and negative control methods were used for quality control, with 5% of samples being randomly selected for duplicate testing, achieving a 100% concordance rate.

### Construction of tissue microarray (TMA) and IHC

Histone modification markers and protein levels were evaluated using TMAs and IHC [16]. TMAs were proceed through a series of steps, including antigen retrieval, blocking and incubating with specific antibodies. IHC stained sections were then digitally captured using the Pannoramic Scanner and analyzed with CaseViewer software. Staining intensity (scored from 0 to 3) and the percentage of positive strained tumor cells (0%–100%) were evaluated by an experienced pathologist blinded to the clinical data.

### Serological tests

Serum cytokine levels were quantified using the Bio-Plex Pro Human Cytokine 27-plex assay (Bio-Rad, M500KCAF0Y) on a Luminex 200 platform (Luminex Corporation, Austin, TX, USA), allowing for rapid and accurate multiplex detection.

IgA and IgG antibodies against EBV VCA-p18 and EBNA-1 were measured using enzyme-linked immunosorbent assay (ELISA) kits (Zhongshan Bio-Tech, Zhongshan, China) [17]. The assays were standardized using a reference serum provided with each kit, with defined optical density (OD) cut-off values determining seropositivity [18].

Anti-*T. gondii* IgG was measured using commercial ELISA kits (Haitai Biological Pharmaceuticals Co., Ltd, Zhuhai, China) [19], with results validated against positive, negative, and critical controls. Similarly, anti-*C. trachomatis* IgG and total IgG were detected using ELISA kits (Savyon diagnostics, Israel and Cusabio Biotech Co, China, respectively), with seropositivity defined as a cut-off index (COI) higher than 1.1. All serological assays were conducted according to the manufacturer’s instructions, with blinded assessment to prevent bias in the analysis of case–control differences.

## What has been found?

### Guangzhou breast *cancer* cohort

Up to 31 December 2023, the cohort has been followed up for a mean of 8.22 years (standard deviation = 3.12). The mean age of participants were 47.9 years at baseline, primarily includes pre-menopausal women (62.8%) and women with menarche after 12 years of age (86.9%) (Table 4). Most participants were married (92.1%) and had a history of breastfeeding (85.8%). Notably, 15% of patients had a family history of BC, 24.3% were overweight or obese (BMI ≥ 25 kg/m^2^ ), and 20.7% were diagnosed at clinical stages III or IV. After a mean follow-up duration of 8.22 years, disease progression was observed in 731 patients (18.2%), and 406 deaths (10.1%) were recorded (Table 5).

**Table 4:** Baseline characteristics of the Guangzhou Breast Cancer Study (GBCS) participants

Characteristics	Guangzhou breast cancer cohort	Case–control study			IHC cohort
	BC patients (n = 5471) (%)	Cases (n = 1551) (%)	Controls (n = 1605) (%)	P*	BC patients (n = 1063) (%)
Age at diagnosis, years	47.92 ± 10.74	48.39 ± 11.46	48.35 ± 11.38		48.46 ± 10.40
≤ 40	1370 (25.0)	419 (27.0)	444 (27.7)		254(23.9)
41–60	3394 (62.1)	907 (58.5)	931 (58.0)		686(64.6)
≥ 61	703 (12.9)	225 (14.5)	230 (14.3)	0.919	122(11.5)
Menopausal status					
Pre-menopausal	3344 (62.8)	928 (60.8)	864 (54.9)		597 (59.3)
Post-menopausal	1985 (37.2)	598 (39.2)	710 (45.1)	< 0.010	409 (40.7)
Educational level					
Below junior school	2469 (48.1)	736 (49.9)	596 (38.2)		294 (55.9)
Senior high school	1284 (25.0)	399 (27.1)	581 (37.2)		116 (22.1)
College or above	1384 (26.9)	340 (23.1)	385 (24.6)	< 0.010	116 (22.1)
Age of menarche, years					
≤ 12	700 (13.1)	194 (13.0)	233 (14.9)		89 (8.7)
> 12	4633 (86.9)	1302 (87.0)	1327 (85.1)	0.117	934 (91.3)
Marital status					
Never married	180 (3.4)	64 (4.2)	62 (4.0)		19 (1.8)
Married	4904 (92.1)	1350 (89.5)	1386 (89.3)		978 (94.7)
Widowed or divorced	239 (4.5)	95 (6.3)	104 (6.7)	0.857	36 (3.5)
Parity					
0	333 (6.2)	125 (8.2)	117 (7.4)		39 (3.8)
≥ 1	5057 (93.8)	1391 (91.8)	1454 (92.6)	0.410	979 (96.2)
BMI, kg/m^2^					
< 23	2848 (53.9)	819 (54.8)	871 (56.7)		514 (51.3)
23–24.9	1152 (21.8)	302 (20.2)	308 (20.0)		234 (23.4)
≥ 25	1281 (24.3)	374 (25.0)	358 (23.3)	0.490	254 (25.3)
Breastfeeding history					
No	725 (14.2)	236 (16.8)	285 (18.7)		53 (9.9)
Yes	4396 (85.8)	1166 (83.2)	1236 (81.3)	0.179	485 (90.1)
Family history					
No	4516 (85.0)	1451 (96.5)	1509 (97.0)		921 (89.5)
Yes	795 (15.0)	52 (3.5)	47 (3.0)	0.493	108 (10.5)
Clinical stage					
I/II	3866 (79.3)	–	-		708 (69.9)
III/IV	1010 (20.7)	–	-		305 (30.1)
ER status					
Negative	1211 (24.3)	–	-		268 (26.5)
Positive	3765 (75.7)	–	-		744 (73.5)
PR status					
Negative	1664 (33.5)	–	–		275 (27.2)
Positive	3297 (66.5)	–	–		735 (72.8)
HER2 status					
Negative	2616 (54.7)	–	–		554 (61.6)
Equivocal	1160 (24.3)	–	–		151 (16.8)
Positive	1006 (21.0)	–	–		194 (21.6)
TNBC					
No	4271 (89.8)	–	–		826 (92.3)
Yes	485 (10.2)	–	–		69 (7.7)
Ki-67					
≤ 14%	1209 (26.4)	–	–		364(36.4)
> 14%	3375 (73.6)	–	–		637(63.6)
Histological grade					
I/II	–	–	–		710 (73.5)
III	–	–	–		256 (26.5)

^*^P values are for differences between cases and control in the case–control study

*BC* Breast cancer, *IHC* Immunohistochemistry

**Table 5:** Follow-up results of Guangzhou breast cancer cohort and immunohistochemistry (IHC) cohort

	Guangzhou breast cancer cohort (n = 4023)	IHC cohort (n = 1048)
Mean follow-up year (SD)	8.22 (3.12)	8.53 (4.02)
Overall mortality, n (%)	406 (10.1%)	189 (18.0%)
Disease progression, n (%)	731 (18.2%)	270 (25.8%)
SD: Standard deviation		

### Case–control study

In the case–control study, participants had a mean age of 48.4 years (Table 4). A higher proportion of controls (45.1%) were post-menopausal compared to cases (39.2%). Education attainment was significantly higher in controls than in cases (P < 0.01), whereas other characteristics showed no significant differences (P > 0.05).

### Immunohistochemistry (IHC) cohort

The IHC cohort, with a mean age of 48.5 years, predominantly comprised women aged 41 to 60 years (64.6%) (Table 4). The distribution of menopausal status, age at menarche and marital status among the participants closely resembled those of the Guangzhou breast cancer cohort. A family history of BC was reported by 10.5% of participants and 21.6% of patients tested positive for HER2 status. 73.5% of patients had a histological grade of I or II. During a mean follow-up of 8.53 years (standard deviation = 4.02), disease progression occurred in 270 (25.8%) patients, with 189 deaths (18.0%) recorded (Table 5).

### Published results

**Fig. 2:**
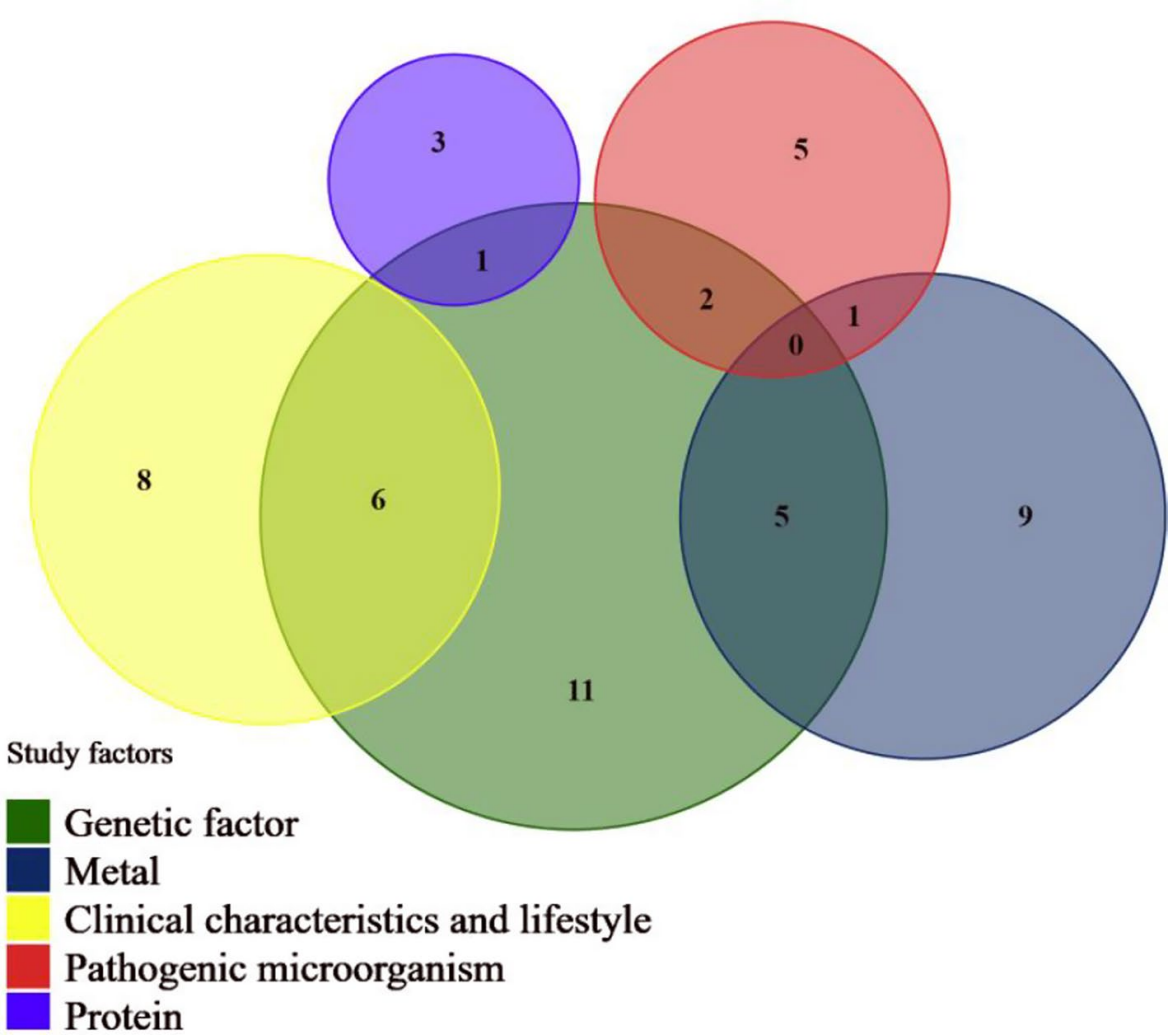
A wayne diagram of factors associated with breast cancer risk and mortality that have been studied by the GBCS*. *The size of the circle represents the number of published articles. The color of the circle represents different categories of research factors

Until to 31 August 2024, the GBCS has published 51 papers on various risk factors related to the occurrence and prognosis of breast cancer, as detailed in Fig. 2 and Supplementary Table 2. Of various factors studies (i.e., genetic factors, metal exposure, clinical characteristics, lifestyles, pathogenic microorganisms and proteins), genetic factors have been a primary focus, with polymorphisms in genes such as *PARP1, ESR1, FGFR2, FGF1, RBFOX2, FTO, IL-6,* and *HSPD1* identified as modifiers of BC risk and survival [20–23]. Epigenetic studies within the cohort showed that histone modifications, including H3K9me2, H3K9me3, H3K4me2, H3K27me3, H4K20me3, and H4K16ac, were strongly associated with disease progression and mortality [24–27].

The associations between urine levels of metal exposure and breast cancer risk have also been evaluated, with findings indicating that higher levels of strontium [odds ratio (OR) (95% confidence interval (CI)) for the highest versus the lowest tertile: 2.24 (1.42–3.81)] [10] and cadmium [OR (95% CI): 1.16 (1.01–1.34)] [28] were associated with a higher risk of BC. Conversely, higher selenium [OR (95% CI): 0.50 (0.30–0.81)] [28], thallium [OR (95% CI): 0.36 (0.21–0.60)] [29], vanadium [OR (95% CI): 0.60 (0.37–0.97)] [29], and cesium levels [ORs (95% CI): 0.50 (0.30–0.82)] [30] were associated with a lower risk of BC, which were consistent with other studies [31–33].

Futhermore, we found that long interval (> 5 years) between first and second birth was associated with a better progression free survival (PFS) [hazard ratio (HR) (95% CI): 0.64 (0.42–0.97)] [34], whereas weight loss at 2 years [HR (95% CI): 1.34 (0.87–2.06)] or more than 2 years [HR (95% CI): 1.95 (1.22–3.10)] after diagnosis increased the risk of BC progression [35]. In addition, specific Chinese lifestyles such as taking a nap [36] and drinking tea [37] appeared to be associated with a lower risk of BC progression.

Infectious agents such as Epstein-Barr virus and *Toxoplasma gondii* infection have also been examined, with results showing that Epstein-Barr virus infection being associated with a higher BC risk among ER + , PR + , and HER2 + patients [18], while *Toxoplasma gondii* infection being associated with a lower BC mortality and progression risk [HR (95% CI):0.60 (0.37–0.99) for mortality; HR (95% CI):0.67 (0.46–0.98) for progression] [38]. Specific mechanisms have been demonstrated in cellular experiments [39, 40].

Additionally, the role of proteins such as FOXA1 in BC prognosis has been characterized, with time-varying effect observed. Specifically, higher FOXA1 expression was initially associated with improved survival rate in the early post-diagnosis period. However, the potential protective effect appeared to diminish over time, eventually giving way to an adverse effect in the later years [16].

### What are the main strengths and weaknesses?

The GBCS represents a unique and comprehensive study in South China, with several strengths. First, the GBCS cohort includes three sub-studies, each focused on investigating distinct aspects of BC, including risk factors, underlying mechanisms and prognostic indicators. This multifaceted approach enables the investigation of BC across the entire disease spectrum, from preventive to treatment. Another strength lies in its extensive and detailed data collection by face-to-face interview using validated questionnaires. This approach captures region-specific risk factors such as socioeconomic conditions, diet habits (tea intake) and lifestyle factors (late sleep timing and napping) at baseline and follow-up. Third, the robust biorepository, which includes blood, urine, and breast carcinoma tissue samples were collected and stored according to standardized protocols, provides valuable resources for future research. This biobank supports advanced analyses such as Mendelian randomization and multi-omics approaches, potentially offering insights into the causal pathways and therapeutic responses in BC. Fourth, the long-term sustainability of the GBCS is supported by continuous funding from the Natural Science Foundation of China and the Science and Technology Planning Project of Guangdong Province, as well as strong institutional collaborations. These factors have contributed to high follow-up rates and minimized attrition. Finally, the extended follow-up period has resulted in a large number of mortality cases, offering opportunities to examine the long-term impacts of risk factors on BC prognosis. However, there are limitations to consider. First, the reliance on self-reported data for lifestyle factors and medical history introduces the potential for recall bias. Additionally, the participants were recruited from three leading hospitals in Guangzhou rather than through random sampling, which may limit the generalizability of findings to other populations. However, random sampling in a hospital-based patient cohort is generally impractical and unlikely to be feasible.

### Can I get hold of the data? Where can I find out more?

The GBCS seeks and encourages collaboration to maximize the use of research data from all over the world. While the dataset contains sensitive information and is not publicly available for download, researchers with specific ideas or proposals are invited to request access. To initiate a collaboration, interested parties should contact the corresponding author, Professor Lin Xu at xulin27@mail.sysu.edu.cn. Further details about the study and opportunities for collaboration can also be obtained through this contact. 

## Supplementary Information

Below is the link to the electronic supplementary material.

Supplementary file1 (DOCX 85 kb)
