# Supplementary material for: Cohort profile: Guangzhou breast cancer study (GBCS)
Source: Eur J Epidemiol. 2024 Dec 16;39(12):1401–10. doi: 10.1007/s10654-024-01180-y (PMC11680655; doi:10.1007/s10654-024-01180-y)
Supplement: Supplementary file 1 — Supplementary file1 (DOCX 85 kb) [file 10654_2024_1180_MOESM1_ESM.docx]

| **Supplementary table 1.** Basic information of 9,029 subjects in the Guangzhou Breast Cancer Study (GBCS). | |
| --- | --- |
| **Classification of disease** | **Case (n=9,029) (%)** |
| Invasive breast cancer | 5136 (56.9) |
| Ductal carcinoma in situ | 335 (3.7) |
| Benign breast disease | 2200 (24.4) |
| Borderline phyllodes tumor | 42 (0.5) |
| Non-breast cancer | 1146 (12.7) |
| Excluded due to kinship | 35 (0.4) |
| Excluded due to another cancer diagnosis | 43 (0.5) |
| Male breast cancer | 1 (0.0) |
| Not specified | 91 (1.0) |

**Supplementary table 2**. Research findings about Guangzhou Breast Cancer Study (GBCS).

| **Topics** | **Title** | **Sample size** | **Findings** |
| --- | --- | --- | --- |
| Genetic factors | AGR2 and FOXA1 as prognostic markers in ER-positive breast cancer [^20^](#_ENREF_20) | 915 | Among ER-positive patients, the poor prognostic role of the high level of FOXA1 was significant only in patients with the low level of AGR2. |
|  | Allelic Expression Imbalance Polymorphisms in Susceptibility Chromosome Regions and the Risk and Survival of Breast Cancer [^12^](#_ENREF_12) | Cohort=1168;  Cases=839  Controls=863 | Suggestive associations of AEI polymorphisms with breast cancer risk (MUC16 rs2591592 and SLAMF1 rs1061217) and prognosis (ZNF331 rs8109631 and CHRAC1 rs10216653). |
|  | Association of Enolase-1 with Prognosis and Immune Infiltration in Breast Cancer by Clinical Stage [^21^](#_ENREF_21) | 961 | ENO1 may be a promising target for precision immunotherapy in breast cancer. |
|  | Association of H3K9me3 with breast cancer prognosis by estrogen receptor status [^22^](#_ENREF_22) | 917 | The high level of H3K9me3 was associated with a better prognosis among ER-positive tumors, particularly ER-high positive tumors. |
|  | Combined low levels of H4K16ac and H4K20me3 predicts poor prognosis in breast cancer [^23^](#_ENREF_23) | 958 | There was an interaction between H4K16ac and H4K20me3 on the prognosis of breast cancer and the combination of them was a superior prognostic marker compared to the single one. |
|  | Decelerated DNA methylation age predicts poor prognosis of breast cancer [^24^](#_ENREF_24) | 1076 | DNAm age of breast cancer tissue, which associated with menopausal status and pathological features, was a strong independent predictor of the prognosis. |
|  | Effects of Infection-Induced Fever and the Interaction with IL6 rs1800796 Polymorphism on the Prognosis of Breast Cancer [^25^](#_ENREF_25) | 4223 | Infection-induced fever was beneficial to breast cancer survival, particularly for women who were the CC genotype of IL6 rs1800796 or node positive. |
|  | H4K20me3, H3K4me2 and H3K9me2 mediate the effect of ER on prognosis in breast cancer [^26^](#_ENREF_26) | 1045 | H4K20me3, H3K4me2 and H3K9me2 mediated the relationship between ER and breast cancer prognosis. |
|  | Modification effects of genetic polymorphisms in FTO, IL-6, and HSPD1 on the associations of diabetes with breast cancer risk and survival [^27^](#_ENREF_27) | Cohort=1168;  Cases=1551  Controls=1605 | The contribution of diabetes to breast cancer risk might be modified by IL-6 rs1800796 and HSPD1 rs2605039. Diabetes and HSPD1 rs2605039 might also influence breast cancer prognosis. |
|  | Polymorphisms in homologous recombination repair genes and the risk and survival of breast cancer [^28^](#_ENREF_28) | Cohort=1374;  Cases=1551  Controls=1605 | The polymorphisms in HRR genes were associated with risk (RFC1 rs6829064) and prognosis (NRM rs1075496 and LIG3 rs1052536). |
|  | Prognostic value of glutaminase 1 in breast cancer depends on H3K27me3 expression and menopausal status [^29^](#_ENREF_29) | 962 | The prognostic effects of GLS on breast cancer correlated to the expression level of H3K27me3 and menopausal status, which would help optimize the medication strategies of GLS inhibitors. |
|  | Survival is associated with repressive histone trimethylation markers in both HR-positive HER2-negative and triple-negative breast cancer patients [^30^](#_ENREF_30) | 914 | H3K9me3 and H3K27me3 were the potential prognostic markers for breast cancer patients with HR-positive/HER2-negative subtype. Importantly, H4K20me3 was a robust prognostic marker for both HR-positive/HER2-negative and TNBC patients. |
|  | Time‑varying effects of FOXA1 on breast cancer prognosis [^31^](#_ENREF_31) | 1041 | This study revealed an independent time-varying effect of FOXA1 on breast cancer prognosis, which would provide an insight into the roles of FOXA1 as a marker of breast cancer prognosis and may help optimize the medication strategies. |
|  | Association of physical activity and polymorphisms in FGFR2 and DNA methylation related genes with breast cancer risk [^32^](#_ENREF_32) | Cases=839  Controls=863 | Both a gene-environment (FGFR2-exercise activity) and a gene-gene (FGFR2-MTHFR) interaction on breast cancer risk. |
|  | Associations of polymorphisms in the genes of FGFR2, FGF1, and RBFOX2 with breast cancer risk by estrogen/progesterone receptor status [^33^](#_ENREF_33) | Cases=839  Controls=863 | A greater association of FGF1 rs250108 and RBFOX2 rs2051579 with ER-negative breast cancer. |
|  | Effects of passive smoking on breast cancer risk in pre/post-menopausal women as modified by polymorphisms of PARP1 and ESR1 [^34^](#_ENREF_34) | Cases=839  Controls=863 | The risk of breast cancer from passive smoking may be influenced by genetic factors, and that the association may differ depending on menopausal status. |
|  | Genetic variants in EBV reactivation-related genes and the risk and survival of breast cancer [^35^](#_ENREF_35) | Cases=1168  Controls=1605 | The variant alleles of TSG101 rs2292179 and ATF2 rs3845744 were associated with a reduced risk of breast cancer, particularly for subjects with BMI <24 (kg/m^2^) and postmenopausal women, respectively. |
|  | Joint effects of Epstein-Barr virus and polymorphisms in interleukin-10 and interferon-γon breast cancer risk [^36^](#_ENREF_36) | Cases=354  Controls=504 | EBV may contribute to the risk of BC and that this contribution may be modified by genetic variations in IFN-γ. |
|  | Joint effects of febrile acute infection and an interferon-γpolymorphism on breast cancer risk [^37^](#_ENREF_37) | Cases=839  Controls=863 | This study indicated a possible link between febrile acute infection and a decreased risk of breast cancer, and this association was modified by IFN-γrs2069705. |
|  | Relation between PARP-1 Val762Ala Polymorphisms and Susceptibility to Breast Cancer [^38^](#_ENREF_38) | Cases=839  Controls=863 | Val762Ala is not obviously correlated with susceptibility to breast cancer. The PARP-1 Val762Ala polymorphisms may not play a role in the etiology of breast cancer. |
|  | Interaction between body mass index and glutamyl cysteine synthase catalyzed subunit gene polymorphisms on breast cancer risk in women [^39^](#_ENREF_39) | Cases=839  Controls=863 | Increased BMI at 20 years may be a protective factor for premenopausal breast cancer; GCLC rsl7883901 was not significantly associated with breast cancer risk, but its variant genotypes significantly increased the risk of breast cancer with current obesity status (BMI≥25 kg/m2). |
| Metal exposure | Association of urinary cesium with breast cancer risk [^40^](#_ENREF_40) | Cases=240  Controls=246 | Cesium may have anticancer efficacy and urinary cesium has potential as a biomarker for breast cancer risk assessment. |
|  | Joint effects between urinary selenium and polymorphisms in methylation related genes on breast cancer risk [^41^](#_ENREF_41) |  | Selenium was associated with a decreased risk of breast cancer and this beneficial effect was limited to women with CC genotype of MTHFR rs1801133. |
|  | Modified effect of urinary cadmium on breast cancer risk by selenium [^42^](#_ENREF_42) |  | The association of urinary cadmium with breast cancer risk was modified by urinary selenium. |
|  | Urinary rubidium in breast cancers [^43^](#_ENREF_43) |  | The urinary levels of rubidium were significantly and inversely associated with risk of breast cancer and had potential to be a biomarker for breast cancer risk assessment. |
|  | Urinary strontium and the risk of breast cancer: A case-control study in Guangzhou, China [^10^](#_ENREF_10) |  | This study suggested a potential role of strontium in the development of breast cancer and urge further studies on the environmental contamination and the physiological and pathological mechanisms of strontium. |
|  | Urinary Titanium and Vanadium and Breast Cancer: A Case-Control Study [^44^](#_ENREF_44) |  | These results have potentially significant implications on nutritional chemoprevention of breast cancer and the development of new anticancer drugs. |
|  | Association between urinary molybdenum and risk of breast cancer [^45^](#_ENREF_45) |  | A moderate level of Molybdenum may have inhibitory effect of breast cancer; while a high level of Molybdenum may increase the risk of breast cancer, especially for postmenopausal women. |
|  | Arsenic and polymorphisms in 5,10 -Methylenetetrahydrofolate reductase and methionine synthase and breast cancer risk [^46^](#_ENREF_46) |  | In this study, we do not find an association between arsenic with breast cancer risk, and there is no modification effect of MTHFR rs1801133 and MTR rs1805087 on the association. |
|  | Modification Effects of Homologous Recombination Repair Gene Polymorphisms on the Associations Between Urinary Metals and Breast Cancer Risk [^47^](#_ENREF_47) |  | This study showed compelling evidence for the interaction between genetic variants within the HRR system and urinary metals on BC risk. |
| Clinical characteristics and lifestyles | Association between weight change and breast cancer prognosis [^48^](#_ENREF_48) | 2016 | Weight loss was detrimental to breast cancer prognosis, particularly for post-menopausal women, while weight gain may be a potential beneficial indicator for the patients with endocrine therapy. |
|  | Associations of reproductive factors with breast cancer prognosis and the modifying effects of menopausal status [^49^](#_ENREF_49) | 3805 | Age at first birth, durations from first/last birth to diagnosis, and intervals between first and second birth should be taken into account when following the patients and assessing the prognosis of breast cancer. |
|  | Effects of tea consumption and the interactions with lipids on breast cancer survival [^50^](#_ENREF_50) | 1551 | Regularly drinking all teas (mainly green tea) except oolong after diagnosis was beneficial to breast cancer survival, particularly for women with normal lipids, while oolong tea may have an impaired effect. |
|  | Interaction of reproductive tract infections with estrogen exposure on breast cancer risk and prognosis [^51^](#_ENREF_51) | Cohort=4264;  Cases=1003  Controls=1107 | Reproductive tract infections may be protective for the initiation and development of breast cancer, particularly for women with a longer interval of lifetime estrogen exposure. |
|  | Joint effects of multiple sleep characteristics on breast cancer progression by menopausal status [^52^](#_ENREF_52) | 1580 | Inadequate sleep duration to feel one's best and poor sleep quality after diagnosis were associated with an increased risk of breast cancer progression, particularly for pre-menopausal women. |
|  | Molecular features in young vs elderly breast cancer patients and the impacts on survival disparities by age at diagnosis [^53^](#_ENREF_53) | 1056 | Compared with middle-aged patients of breast cancer, poorer prognosis of elderly patients may be caused by aging, while poorer prognosis of young patients was probably mediated through intrinsic characteristics, such as basal-like subtype, GATA3 mutations, and DNAm age of the cancerous tissues. |
|  | Patient and Care Delays of Breast Cancer in China [^54^](#_ENREF_54) | 1551 | Patient delay was the main type of delay in Guangzhou and resulted in higher clinical stage and poor prognosis of breast cancer. Screening for breast cancer among premenopausal women may be an effective way to reduce this delay. |
|  | Endocrine therapy for breast cancer and its effects on the prognosis of breast cancer patients [^55^](#_ENREF_55) | 3527 | The proportion of hormone receptor-positive patients with inappropriate endocrine therapy is found to be similar to that reported in other countries and regions |
|  | Night-shift work, sleep duration, daytime napping, and breast cancer risk [^11^](#_ENREF_11) | Cases=712  Controls=742 | Sleep problems, including night-shift work, and shorter and longer sleep duration, are associated with an increased breast cancer risk. In particular, the combined effects of night-shift work with no daytime napping or longer sleep duration are greater than the independent effects. |
| Pathogenic microorganism | Beneficial Effect of *Toxoplasma gondii* Infection on the Prognosis of Breast Cancer Was Modified by Cytokines [^56^](#_ENREF_56) | 1121 | Anti-*T. gondii* IgG was found to be beneficial to breast cancer survival, suggesting the potential of *T. gondii* as a prognostic marker and a novel immunotherapy approach for cancer patients. |
|  | Association of Epstein-Barr virus and passive smoking with the risk of breast cancer among Chinese women [^57^](#_ENREF_57) | Cases=349  Controls=500 | There was no synergic action between passive smoking and EBV IgA seropositivity on the risk of breast cancer. |
|  | Associations of Epstein-Barr Virus DNA in PBMCs and the Subtypes with Breast Cancer Risk [^58^](#_ENREF_58) | Cases=164  Controls=206 | The presence of EBV DNA in PBMCs may not be an appropriate biomarker for breast cancer risk. The subtype D of EBV was likely to be related to breast tumorigenesis. |
|  | Epstein-Barr virus and breast cancer: Serological study in a high-incidence area of nasopharyngeal carcinoma [^18^](#_ENREF_18) | Cases=223  Controls=309 | The EBV association with BC in an endemic area of nasopharyngeal carcinoma. |
|  | Association of *Chlamydia trachomatis* Infection with Breast Cancer Risk and the Modification Effect of IL-12 [^59^](#_ENREF_59) | Cases=1121  Controls=400 | *C. trachomatis* infection may contribute to the development of hormone-responsive breast cancer in women with high levels of IL-12. Further studies are needed to uncover the underlying mechanisms. |
| Review | Research progress on the relationship between Epstein-Barr virus and breast cancer [^60^](#_ENREF_60) |  | Reviewed the studies on EBV biology and the association of EBV with breast cancer, including EBV detection in breast cancer tissues, serological tests, cytologic experiments and clinical analyses, and described the limitations of current studies and future directions. |
|  | Advances in the study of DNA methylation biomarkers associated with breast cancer [^61^](#_ENREF_61) |  | Reviewed the research and application of DNA methylation, the most important epigenetic mechanism, in the diagnosis, treatment and prognosis of breast cancer. |
| Mechanism | Differential epigenetic and transcriptional profile in MCF-7 breast cancer cells exposed to cadmium [^62^](#_ENREF_62) |  | Cd epigenetically regulated several pathways involving in breast carcinogenesis, particularly the Wnt signaling and metabolic pathways, among which TXNRD1 and CCT3 might play critical roles. |
|  | Differential epigenetic profiles induced by sodium selenite in breast cancer cells [^63^](#_ENREF_63) |  | These results confirmed the epigenetic effects of sodium selenite and revealed the epigenetic profiles in breast cancer cells, which would help understand the mechanisms of Se against breast cancer. |
|  | Identification of epigenetic modifications mediating the antagonistic effect of selenium against cadmium-induced breast carcinogenesis [^64^](#_ENREF_64) |  | Se antagonized the Cd-induced breast carcinogenesis via epigenetic modification and revealed the critical pathways. |
|  | Metal/metalloid levels and variation in lifetime cancer risks among tissues [^65^](#_ENREF_65) |  | Cd-based metal/metalloid levels may be an explanation for the variation in lifetime cancer risk among tissues. Furthermore, the analytical strategy may in turn help identify environmental factors involved in cancer etiology. |
|  | *Toxoplasma gondii* suppresses proliferation and migration of breast cancer cells by regulating their transcriptome [^66^](#_ENREF_66) |  | *T. gondii* transcriptionally regulates several signaling pathways by altering the hub genes such as BRCA1, MYC and IL-6, which can inhibit the breast tumor growth and migration, hinting at a potential therapeutic strategy. |
| Cross-sectional  study | Risk assessment of breast cancer in Guangdong, China: a community-based survey [^67^](#_ENREF_67) |  | Family history of breast cancer, X-ray received benign breast disease and hyperlipoidemia or hypercholesteremia were significantly associated with risk of breast cancer and may have potential for breast cancer risk assessment. |
|  | Correlation between urinary cadmium level and clinicopathological characteristics of breast cancer [^68^](#_ENREF_68) |  | Urinary cadmium level was associated with HER-2 status, lymph node and/or distant metastasis and TNM stage of breast cancer patients. Cadmium may cause aggressive and malignant human breast cancer. |
|  | Expression of HER-2, p53, estrogen receptor and progesterone receptor in tissues of invasive ductal breast carcinoma with different clinical stages [^69^](#_ENREF_69) |  | Expression of HER2 and PR might be better markers for predicting clinical stages and prognosis. |
